# Pregnancy and Cancer Uteri

**Published:** 1844-07-27

**Authors:** 


PREGNANCY AND CANCER UTERI.

Dr. Miller, jr. narrates, in the London and Edinburg Medical Journal, a melancholy case of this complication, in which the poor woman died undelivered. On examination of the body, the fœtus was found to have been dead several days; the uterus was deeply involved in the carcinomatous degeneration, and partly destroyed by ulceration. The ovaries were also altered in structure, having a soft caseous consistence, and breaking down easily under the finger. The left ovary was nearly twice as large as the right, which was about the normal size. Dr. Miller suggests the induction of premature labour in future in cases of scirrhus uteri as soon as the existence of pregnancy can be ascertained, as the best means of prolonging the life of the patient.

